# Sugar-Based Monoester Surfactants: Synthetic Methodologies, Properties, and Biological Activities

**DOI:** 10.3390/antibiotics12101500

**Published:** 2023-09-30

**Authors:** Michele Verboni, Diego Romano Perinelli, Alessandro Buono, Raffaella Campana, Maurizio Sisti, Andrea Duranti, Simone Lucarini

**Affiliations:** 1 Department of Biomolecular Sciences, University of Urbino Carlo Bo, 61029 Urbino, Italy; michele.verboni@uniurb.it (M.V.); a.buono6@campus.uniurb.it (A.B.); raffaella.campana@uniurb.it (R.C.); maurizio.sisti@uniurb.it (M.S.); simone.lucarini@uniurb.it (S.L.); 2 School of Pharmacy, University of Camerino, Via Gentile III da Varano, 62032 Camerino, Italy; diego.perinelli@unicam.it

**Keywords:** glycolipids, sugar-based surfactants, enzymatic synthesis, green chemistry, permeability enhancers

## Abstract

Glycolipids are biocompatible and biodegradable amphiphilic compounds characterized by a great scientific interest for their potential applications in various technological areas, including pharmaceuticals, cosmetics, agriculture, and food production. This report summarizes the available synthetic methodologies, physicochemical properties, and biological activity of sugar fatty acid ester surfactants, with a particular focus on 6-*O*-glucose, 6-*O*-mannose, 6-*O*-sucrose, and 6′-*O*-lactose ones. In detail, the synthetic approaches to this class of compounds, such as enzymatic lipase-catalyzed and traditional chemical (e.g., acyl chloride, Steglich, Mitsunobu) esterifications, are reported. Moreover, aspects related to the surface activity of these amphiphiles, such as their ability to decrease surface tension, critical micelle concentration, and emulsifying and foaming ability, are described. Biological applications with a focus on the permeability-enhancing effect across the skin or mucosa, antimicrobial and antifungal activities, as well as antibiofilm properties, are also presented. The information reported here on sugar-based ester surfactants is helpful to broaden the interest and the possible innovative applications of this class of amphiphiles in different technological fields in the future.

## 1. Introduction

Amphiphilic compounds possess both hydrophilic and lipophilic properties and are termed SURFace, ACTive, AgeNTS, or SURFACTANTS. They can be ionic (anionic, cationic, and amphoteric) or neutral molecules and the most globally synthesized surfactants are linear alkylbenzene sulphonates, α-olefin sulphonates, alcohol ether sulphates, fatty alcohol, and alkylphenol ethoxylates, polysorbates, etc. [1]. Although these compounds are widely used, most of them are toxic and dangerous for the environment due to their recalcitrant and persistent nature [2]. Current research is focused on alternative environmentally friendly natural-surfactants or biosurfactants. In this field, sugar fatty acid esters, usually called sugar-based esters (SBEs), represent an important class of “green” non-ionic surfactants. In fact, they are non-toxic, biodegradable, non-irritant, odorless, and have a safe biocompatibility profile [3]. SBEs are constituted by a carbohydrate moiety (mono, di- or oligosaccharide) linked by an ester bond to one or more fatty acid chains. Some SBEs are naturally obtained in plants but as mixtures at low concentrations, thus resulting in very expensive extraction processes [4,5]. Moreover, they can be produced in huge amounts from cheap raw materials (oily and industrial by-products) by microorganism fermentation processes [6]. On the other hands, these compounds can be easily synthesized by enzymatic or chemical esterification reactions, using the corresponding carbohydrates (e.g., sucrose from beet or lactose from milk and derivatives) and fatty acids (from several natural origin such as vegetal oils, dry fruits, and animal fats) [7,8].

Amongst the SBEs produced globally, sugar-based monoesters such as glucose and sucrose have been the most studied and commercially used, while mannose, lactose, and other monoester derivatives have attracted great interest from the scientific community in the last decade due to their promising properties and biological activities [9,10]. Most of the time, these compounds are excellent emulsifiers and dispersing and foaming agents, showing very good solubilization, wetting, and detergent capabilities, and are produced from inexpensive and renewable products [11]. Sugar-based monoesters also have good antibacterial, antifungal, and insecticidal activities, and permeability-enhancing properties [12]. For all these reasons, they are already necessary compounds in the nutraceutical, food, cosmetic, and pharmaceutical industries. Due to their applications, in the 2010s the world production of only sucrose fatty esters was above 6.000 tons per year and their market is projected to grow from USD 76 million in 2019 to USD 106 million by 2025 [13].

**Figure 1:**
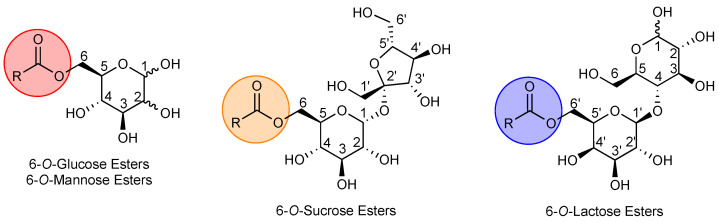
General Structural Formulas of Sugar-based Monoesters.

This report presents the synthesis, properties, and biological applications of 6-*O*-glucose, 6-*O*-mannose, 6-*O*-sucrose and 6′-*O*-lactose monoesters (Figure 1).

In detail, recently regioselective enzymatic and chemical esterification reactions were described. Moreover, a brief focus on the surface-active properties linked to the critical micelle concentration of these monoesters was highlighted, followed by their foaming and emulsifier capacities. Subsequently, numerous studies of their ability to act as permeability enhancers were conducted. Finally, the evaluation of their antimicrobial activities against Gram-positive, Gram-negative, and fungi, together with their antibiofilm activities, were widely discussed.

## 2. Synthesis of Sugar-Based Esters

In recent years a series of different SBEs formed by carbohydrates coupled with fatty acids or alkyl aromatic and aromatic acids have been synthesized, such as 6-*O*-glucose esters (GEs), 6-*O*-mannose esters (MEs), 6-*O*-sucrose esters (SEs), and 6′-*O*-lactose esters (LEs) (Figure 1).

**Figure 2:**
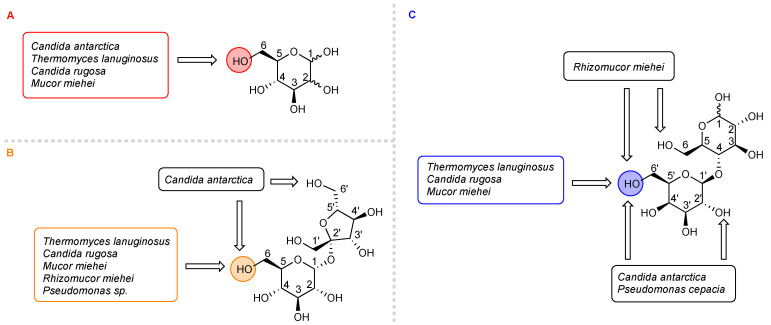
Lipase Regioselectivity at 6-OH-Glucose, -Mannose (**A**), and -Sucrose (**B**), and 6′-OH-Lactose (**C**).

Commonly, SBEs can be synthesized using renewable and sustainable materials (carbohydrate and fatty acids) by simple chemical or enzymatic reactions [14], while their properties can be easily tuned by the degree of substitutions, the type and length of the fatty acid, and the nature of the sugar moiety [15]. On the other hand, the multiple stereogenic centers and hydroxyl groups of the carbohydrates (e.g., eight in sucrose and lactose) and their low solubility in green solvents represent major problems for obtaining SBEs in a regioselective manner. Chemical synthesis is the most commonly used in the industry for the large-scale production of SBEs at low costs and high yields. However, the chemical esterification usually requires high temperatures and toxic acylating agents (e.g., acyl chlorides) and solvents ([e.g., pyridine, dimethyl sulfoxide (DMSO))] [16]. Moreover, chemical glycolipid reactions are usually low-regioselective, leading to a mixture of mono-, di-, and polyacylated sugar esters. Therefore, the installation of protective groups on sugar moieties is often adopted [17]. On the other hand, regioselective esterification can often be achieved by enzymatic reactions mainly using lipases, which are biodegradable and versatile enzymes characterized by their stability under different conditions (pH, wide range of temperatures, and active multiple organic solvents) [18]. Lipase-catalyzed reactions require fatty acids or their vinyl ester analogues as acyl donors instead of acyl chlorides [19], and they are usually carried out under mild conditions with eco-friendly solvents (e.g., acetonitrile, ethanol, or *tert*-butanol). Notably, immobilized lipases are stable in various reaction conditions and can be recovered and reused for other reaction cycles without significant loss of activity [20]. However, the residual content of water affects the formation of ester products; for this reason, the use of anhydrous solvents is necessary. Moreover, molecular sieves are usually added to the medium of the reaction to trap the water produced during the lipase-catalyzed esterification reaction and, consequently, to efficiently enhance the sugar ester formation. Most of the lipases used for sugar ester synthesis in non-aqueous environments have a microbial origin [21]. The lipases mostly used for regioselective esterification in the desired primary position of glucose, mannose, sucrose, and lactose are from *Candida antarctica*, *Candida rugosa*, *Rhizomucor miehei*, *Thermomyces lanuginosus*, *Mucor miehei*, *Pseudomonas cepacia*, and *Pseudomonas* sp. (Figure 2). The drawbacks of enzymatic reactions in SBEs are represented by the high cost of enzymes, long reaction time, and modest yields. Below, the synthetic procedures adopted for the synthesis of GEs, MEs, SEs, and LEs developed by our research group and other authors are reported.

### 2.1. Synthesis of 6-*O*-Glucose Esters and 6-*O*-Mannose Esters

**Scheme 1:**
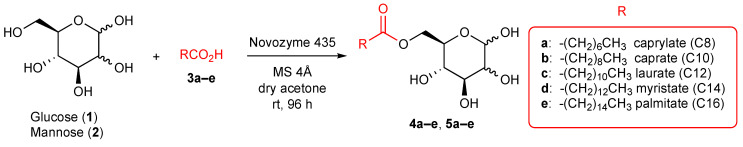
Synthesis of 6-*O*-Glucose and 6-*O*-Mannose Esters (**5a**–**e**), Catalyzed by Novozyme 435.

GEs (**4a**–**e**) and MEs (**5a**–**e**) derivatives were synthesized using a reported catalyzed reaction [22] (Scheme 1). Glucose (**1**) and mannose (**2**) were coupled with saturated fatty acids C8–C16 (**3**) in a 1:3 molar ratio and in the presence of Novozyme 435 (from *C. antarctica* immobilized on acrylic resin) and molecular sieves 4 Å in dry acetone at room temperature for 96 h [23]. This reaction has the advantage of producing **4a**–**e** and **5a**–**e** in a regioselective manner and has easy purification steps. These glycolipids were obtained as a mixture of α/β anomers in good yields.

Several lipases (*C. antarctica*, *C. rugosa*, *T. lanuginosus*, *M. miehei*)-catalyzed reactions are reported for the regioselective acylation at position 6 of the glucose and mannose, as shown in Figure 2A [24]. Generally, Novozyme 435 (immobilized from *C. antarctica*) is the most applied lipase for the synthesis of monosaccharide esters due to its high activity, stability, and availability at moderate costs. Liang et al. used this lipase to catalyze a facile and irreversible esterification of D-glucose with different vinyl esters (C6–C18) in THF/pyridine 4:1 [25]. Arcens et al. reported the conversion of glucose to glucose-6-*O*-palmitate (C16) operated by Novozyme 435 at 45 °C in acetonitrile for 72 h [26]. In particular, the importance of using dry acetonitrile for achieving a complete conversion was highlighted. With regard to the solvent, ionic liquids represent a good alternative to the common solvents for the enzymatic synthesis of SBEs due to their thermal stability and recyclability [27]. Moreover, ionic liquids have some advantages such as preserving the high activity of lipase and improving both saccharide and acyl donor solubilities. Shin et al. reported a Novozyme 435-catalyzed esterification of glucose with vinyl laurate in a mixture of ionic liquids constituted by [Bmim][TfO]:[Bmim][Tf2N] (1:1 *v*/*v*). It is worth mentioning that the higher solubility observed for glucose and vinyl laurate in ionic liquid mixtures than in other organic solvents leads to a conversion of glucose that is twice as superior [28]. Zhao et al. also investigated glucose transesterification with vinyl esters (C10-C18) using ionic liquids. In this case, the reaction was catalyzed by Lipozyme^®^ TL IM (immobilized from *T. lanuginosus*) in a co-solvent system that constituted 2-methyl-2-butanol (2M2B) and 1-hexyl-3-methylimidazolium trifluoromethylsulfonate ([HMIm][TfO]) [29]. With concern for synthetic procedures, AlFindee et al. used different acyl chlorides (C2–C16) for the formation of a series of 6-*O*-monosaccharide esters in the presence of acyl chlorides and 4-dimethylaminopyridine (DMAP) in pyridine. It was observed that there was a major regioselectivity at 6-OH-glucose rather than 6-OH-mannose. In fact, mannose esters were obtained in a 4:1 mixture of 6-*O*- and 2-*O*-monoesters [10]. Esterification via acyl chloride was also adopted by Jumina et al. for the synthesis of 6-*O*-glucose myristate (C14) [30]. The reaction proceeds with a 1:3 sugar/myristoyl chloride molar ratio in pyridine at 95 °C for 40 min.

### 2.2. Synthesis of 6-*O*-Sucrose Esters

**Scheme 2:**
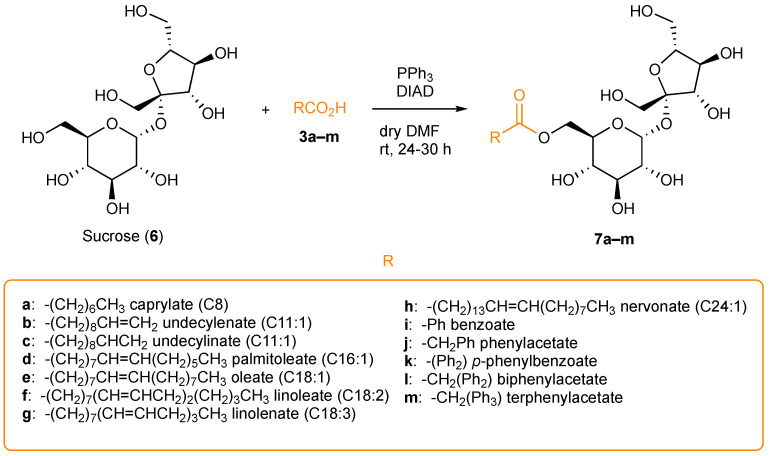
Synthesis of 6-*O*-Sucrose Esters (**7a**–**m**) by a Mitsunobu-type Reaction.

SEs (**7a**–**m**) were synthesized using a modified Mitsunobu procedure [31] starting from sucrose (**6**) and various saturated or (poly)unsaturated fatty acids, or alkyl aromatic/aromatic acids (**3**) (Scheme 2). This optimized reaction gave preferentially the esterification at 6-OH of sucrose and proceeds with a sucrose/fatty acid 1:1.5 molar ratio using 2.5 equivalents of triphenylphosphine (PPh_3_) and diisopropyl azodicarboxylate (DIAD) in dry dimethylformamide (DMF) at room temperature for 24–30 h [32,33]. The procedure gave the corresponding 6-*O*-sucrose esters in moderate yields. However, it is worth mentioning that this reaction was found to be versatile for all the acid substrates (particularly aromatic acids).

Among the glycolipids, sucrose esters are certainly the most studied in regard to their activity and production, whereas about the enzymatic esterification of 6-*O*-acylsucrose esters, the regioselective action of lipases for hydroxyl groups is shown in Figure 2B [7]. More recently, Zhu et al. synthesized a series of 6-*O*-sucrose fatty acid esters (C12-C20) via Lipozyme^®^ TL IM (immobilized from *T. lanuginosus*) using vinyl esters as acyl donors in a mixture of *tert*-butanol/pyridine in a 1:1 mixture [34]. The same lipase was used by Shao et al. for the synthesis of 6-*O*-sucrose laurate (C12) from vinyl laurate in the mixture of ionic liquid-organic solvent [3CIM(EO)][NTf_2_] and 1.5:1. This ionic liquid can improve the solubility of the reagents and is able to sustain the activity of the lipase [35]. Supercritical CO_2_ was also employed as a solvent in various lipase-catalyzed reactions due to its non-toxic and non-flammable nature. For example, Habulin et al. synthesized 6-*O*-sucrose laurate (C12) using Novozyme 435 and supercritical CO_2_, obtaining a 74% lauric acid conversion at 60 °C in 24 h [36]. A valid alternative to enzymes could be represented by resins. Particularly, Sasayama et al. used Diaion WA20 for the transesterification of sucrose with methyl oleate [37]. This weak, basic resin showed a very low decomposition of substrates and sucrose esters with respect to the stronger basic resins. In fact, the yield of the product was higher using Diaion WA20 than stronger basic resins, with a sucrose/methyl oleate molar ratio of 12:1. This catalyst could be employed in the large-scale synthesis of SEs. In regard to gram-scale production, an important contribution was made by Xie et al., who reported a new synthetic procedure to obtain a high-pure sucrose monostearate ester (C18) under solvent-free conditions. This approach required a high temperature with the use of methyl stearate/potassium stearate and K_2_CO_3_/KOH as the base catalyst. The final sucrose ester mixtures contain a monoester fraction in the range of 41–75% [38]. With regard to the chemical esterification, the regioselective production of SEs in one step is challenging due to the presence of eight different hydroxyl groups. Generally, different acyl chlorides and other acylating agents (e.g., acyl anhydrides, enol esters) have been utilized [39,40]. In all cases, a mixture of monoacylated and/or diacylated SEs has been obtained.

### 2.3. Synthesis of 6′-*O*-Lactose Esters

**Scheme 3:**
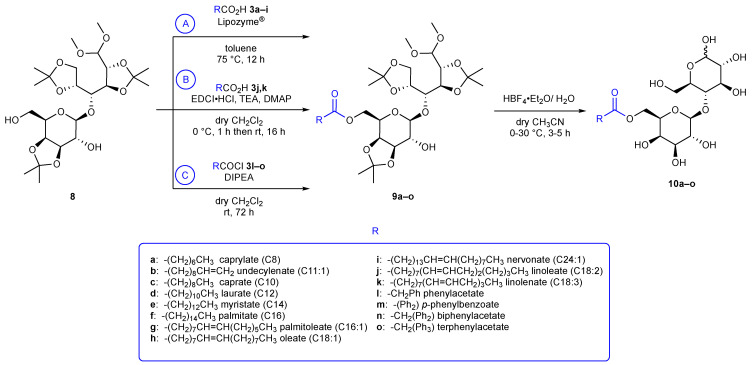
Synthetic Procedures for 6′-*O*-Lactose Esters (**10a**–**o**). (**A**): 6′-*O*-LTA-Fatty Acid Esters by Lipozyme^®^. (**B**): 6′-*O*-LTA-Polyunsaturated Fatty Acid Esters by a Modified Steglich Reaction. (**C**): 6′-*O*-LTA-Alkyl Aromatic or Aromatic Esters Using Acyl Chlorides.

LBEs (**10a**–**o**) were synthesized in a two-step procedure starting from 4-*O*-(3′,4′-*O*-isopropylidene-β-D-galactopyranosyl)-2,3:5,6-di-*O*-isopropylidene-1,1-di-*O*-methyl-D-glucopyranose (lactose tetra acetal, LTA, **8**) (Scheme 3), which was previously obtained from the protection of lactose using 2,2-dimethoxypropane and catalytic *p*-TSA at reflux [41]. The first step concerned the esterification (chemical or enzymatic) of LTA (**8**) with saturated/monounsaturated (**3a**–**i**) or polyunsaturated (**3j**,**k**) fatty acids or alkyl aromatic and aromatic acids (**3l**–**o**). For the synthesis of 6′-*O*-LTA-fatty acid esters (**9a**–**i**), an enzymatic esterification catalyzed by Lipozyme^®^ (immobilized lipase from *M. miehei*) was adopted. In detail, LTA was coupled with the appropriate fatty acid in a 1:1 molar ratio in the presence of Lipozyme^®^ in toluene at 75 °C for 12 h, giving the 6′-*O*-LTA esters regioselectivity in good yields (Scheme 3A) [23,42,43,44]. Notably, Lipozyme^®^ could be recycled at least three times, preserving comparable activity [45]. With regard to the synthesis of 6′-*O*-LTA-polyunsaturated fatty acid esters (**9j**,**k**), a modified Steglich esterification was selected (Scheme 3B) [46]. The reaction involved using a 1:1.2 LTA/fatty acid molar ratio of 1-ethyl-3-(3-dimethylaminopropyl)carbodiimide hydrochloride (EDC∙HCl) instead of *N*,*N’*-dicyclohexylcarbodiimide (DCC), dry triethylamine, and catalytic DMAP in dry dichloromethane at room temperature for 16 h. Despite the modest yields, compounds **9j**,**k** were obtained in a regioselective manner. On the other hand, 6′-*O*-LTA-alkyl aromatic or aromatic esters (**9i**–**o**) were synthesized by conventional esterification using acyl chlorides and *N*,*N’*-diisopropylethylamine (DIPEA) (Scheme 3, pathway C), since Lipozyme^®^ cannot tolerate aromatic acids [23]. It is worth noting that this procedure is regioselective for 6′-OH. The second step was common for all LTA esters (**9a**–**o**) and concerned acidic deprotection by a catalytic amount of tetrafluoroboric acid diethyl ether complex (HBF_4_·Et_2_O) in dry acetonitrile, which led to the desired LBEs (**10a**–**o**). To the best of our knowledge, these are the first synthesis reported for 6′-*O*-lactose polyunsaturated esters and 6′-*O*-lactose alkyl aromatic or aromatic esters.

Figure 2C presents the lipases that catalyzed at the 6′-OH-position (Figure 2C) [12]. For example, Zaidan et al. used NER-CRL (immobilized from *C. rugosa*) for the synthesis of 6′-*O*-lactose caprate (C10), starting from a lactose/capric acid 2:1 molar ratio in acetone [47]. Liang et al. utilized Lipozyme^®^ TL IM to catalyze the synthesis of different LEs (C6–C18) using lactose and 1:3 molar ratio vinyl esters in THF/pyridine 1:1 [48]. Enayati et al. studied the influence of different solvents and free or immobilized lipase CALB (*C. antarctica* lipase B) in the conversion of lauric acid to 6-*O*-lactose laurate (C12) [49]. The best result was obtained with immobilized lipase using hexane, with a 1:1 molar ratio lactose/lauric acid at 50 °C in 12 days. In this study, it was highlighted that the free lipase was highly influenced by the type of solvent than the immobilized ones. In fact, immobilized lipases showed a better conversion associated with less solvent dependency.

As a suitable alternative to lipases, Enayati’s group used aluminosilicate zeolite as Lewis’s acid catalyst for the synthesis of 6-*O*-lactose laurate [49]. Aluminosilicate minerals are available at a low price, and they are produced in high quantities from waste materials, representing a valid alternative to lipases. Moreover, they act as molecular sieves favoring the formation of ester product. The best result was obtained with a 1:2 molar ratio lactose/lauric acid using *tert*-butanol in 10 days. Recently, a nanodendritic metal-magnetic catalyst was applied for the regioselective synthesis of biosurfactants for the first time. In detail, Karami et al. synthesized lactose-6′-*O*-laurate (C12) by a novel metallic encapsulated magnetic core/dendrimer shell composite as a catalyst (Co^II^/Mn^II^ G_2.0_L_1/2_@SCMBNP) [50]. The optimized conditions were obtained with a 2:1 molar ratio lactose/lauric acid at 50 °C in 8 days. Furthermore, the Co^II^/Mn^II^ G_2.0_L_1/2_@SCMBNP could be recovered and reused for five reaction times with only a small loss of catalytic efficiency.

## 3. Surface Active Properties and Critical Micelle Concentration (CMC) Determination

CMC represents the surfactant concentration at which the surface/interface is fully saturated by amphiphiles monolayers and micelle formation occurs in aqueous media, while the surface tension value measured at CMC is referred to as γCMC. These are surface active parameters that are characteristic of pure amphiphiles and describe their behavior in aqueous media [51]. Sugar *O*-monoesters are derived from glucose, mannose, sucrose, and lactose bearing a hydrocarbon chain of at least eight carbon length demonstrated to be good surface-active compounds able to decrease air-water surface tension and saturate the interface at relatively low concentrations. Indeed, CMC values for these surfactants are generally in the range 0.1–40 mM, and, as for other amphiphiles, they are strongly dependent on the length of the hydrophobic chain, with an inverse relationship between the length chain and CMC of the surfactants [34,43]. On the other side, it is not straightforward to assess the effect of the different sugar moiety on CMC for surfactants bearing the same hydrophobic acyl chain due to the lack of direct comparative studies. However, the effect of the sugar moiety, especially whether two disaccharides or monosaccharides are compared, seems to be quite marginal from the available studies [52,53,54]. An et al. synthesized a series of 6-*O*-glucosyl esters with acyl glycinates that were 10, 12, 14 or 16 in carbon chain length as the hydrophobic portion. The calculated CMC values were 12.58, 6.31, 1.75 and 0.77 mM at 25 °C, respectively, while γCMC ranged from ~35 mM/m to 27 mN/m [55]. The CMC values of these glucose-based derivatives seemed to be higher than those reported for 6′-*O*-glucose esters with fatty acids. Indeed, the CMC for 6′-*O*-glucose C10 ester was 2.5 mM [56]. CMC values such as glucose-based surfactants were calculated for the other monosaccharide maltose. The CMC and γCMC of 6′-*O*-maltose C10 ester was 2.25 mM and 27.7 mN/m, respectively [57]. The same paper together with another one also reported the CMC and γCMC of 6-*O*-maltose C14 ester with good agreement (0.018 mM and 27.7 mN/m [57] and 0.02 mM and 27.5 mN/m [58]). Some studies have evaluated the surface properties of homologous series of acyl esters made up of disaccharides as polar heads such as lactose and sucrose. Liang et al. reported the CMC and γCMC of 6′-*O*-lactose esters with C8–C18 saturated acyl chains. The determined CMC values were in the range 1.25–0.012 mM and γCMC between 35 mN/m and 31 mN/m [25]. Another 6′-*O*-lactose C10–C16 ester series was synthesized by Lucarini et al., who reported CMC values from ~2.6 mM to 0.08 mM and γCMC values in the range of 40–45 mN/m [43]. Both studies confirmed the decrease in CMC values with the elongation of the acyl chain, and the more marked effect exerted by the homologation of the acyl chain on γCMC values. A similar trend for the surface parameters (CMC and γCMC) has been reported for sucrose monoesters. For *O*-sucrose C12, a CMC of 0.45 mM and γCMC of ~35 mN/m has been determined in [52], and a CMC of 0.34 mM and a γCMC of ~37 mN/m has been reported in [53]. Recently, Zhu et al. reported the surface tension analysis of different sucrose monoesters with saturated or unsaturated acyl chains. The saturated homologue series (C12–18) showed CMC values in the range 0.21–0.022 mM. Overall, disaccharide monoesters showed CMC values comparable or slightly higher than those of the corresponding monosaccharide monoesters bearing the same acyl chain, underlining the less marked effect of the sugar polar head related to the length of the hydrophobic chain in determining surface parameters such as CMC [34].

## 4. Foaming and Emulsifying Properties

In accordance with the available literature, sugar monoesters displayed foaming properties and the ability to stabilize emulsions. Liang et al. evaluated the foamability (defined as the ratio between the initial foam height and the height of the solution), foam stability (defined as the ratio between the foam height at some time points and the initial foam height), and the emulsification index using canola oil of a series of 6-*O*-glucose C6–C18 monoesters. The better foamability and foam stability was displayed for surfactants with intermediate acyl chains (C10–C12). On the other side, the ability to stabilize an emulsion, expressed as the emulsification index (ESI), increased according to the elongation of the acyl chains and had the highest value for the stearoyl derivative [48].

ESI (%) = A_0_ × 20/[A_0_ − A_20_] × 100 (1)

where A_0_ and A_20_ are the absorbance at 500 nm obtained at 0 and 20 min.

These results are also confirmed by Ren and Lamsal, who showed the higher ESI for glucose C16 than for glucose C12 [59]. Both studies explained the results by considering the stronger hydrophobic interactions exerted by longer alkyl chains with oil droplets. In another study, Liang et al. evaluated the foamability and emulsifying properties of a series of lactose monoesters (C6–C18) using canola oil. A trend similar to that of glucose monoesters was observed for lactose monoesters in terms of foamability and foam stability, since the best performances were achieved for the lactose C12. With regard to the emulsifying ability, differently from glucose monoesters, the highest ESI was calculated for the lactose C14. Therefore, ESI increased, moving from C6 to C14 lactose surfactants, and then, a marked decreased for the lactose C16 and C18 derivatives was observed [48]. The better foamability for medium-chain derivatives was also observed for 6-sucrose monoesters. Indeed, a study reported the higher foamability of sucrose C12 with respect to C10 and C8 homologs [52]. On the other side, the foamability decreased for derivatives with an acyl chain length longer than C14 due to the larger hydrophobicity and higher molecular weight of these derivatives, which reduced their adsorption at the air–water interface [34]. As for the other sugars, the best emulsifying ability for sucrose monoesters was observed for longer derivatives up to a C18 acyl chain. This was demonstrated using different oil phases (sunflower, soybean, olive, and colza oils), both by measuring the ESI parameter [52,60] and following the droplet size distribution of emulsions stabilized with sucrose monoesters having acyl chains of a different length [34,61].

## 5. Sugar Esters as Permeability-Enhancing Compounds

The potential ability of amphiphiles to act as penetration enhancers across the skin or permeability enhancers across the mucosa represents an interesting application for this type of compound. These properties can have a great impact in the field of biopharmaceutics and drug delivery, since they enable the development of novel formulations for the administration of drugs and macromolecular biotherapeutics across non-invasive routes of administration, such as nasal, pulmonary, intestinal, or transcutaneous [62,63,64]. All these routes can be considered alternatives to the direct injection of the therapeutic agent inside the systemic circulation by avoiding some of the disadvantages related to parenteral administration (e.g., poor patient compliance). Among all sugar-based esters, those derived from sucrose have been the most investigated, starting from the late 1990s and early 2000s. Most of the studies carried out in those years focused on the evaluation of the ability to increase the penetration of small molecules as drugs across the skin [65]. More recently, scientific interest has moved to the assessment of the potential ability of sucrose esters to act as permeability enhancers also across the mucosa. The effect on the nasal epithelial permeability of non-cytotoxic concentrations of sucrose C12 and C14 esters was assessed using RPMI 2650 human cell lines. The selected non-toxic concentration (0.1 mg/mL) was able to exert an increase in the permeability of fluorescein isothiocyanate (FITC)-labelled 4.4 kDa dextran across nasal cells via the paracellular route in a concentration-dependent manner [66]. Apart from this, the largest number of studies was devoted to the intestinal route of drug administration. The in vitro ability of sucrose C12, C14, and C16 to reduce the transepithelial electrical resistance (TEER) and increase the permeation of atenolol, fluorescein, vinblastine, caffeine, antipyrine, and rhodamine 123 across Caco-2 cells, as a model for the intestinal route, was assessed by Kiss et al. [67]. This study demonstrated that sugar esters at non-cytotoxic concentrations can promote drug absorption across intestinal model cells through the trans- and paracellular routes without inhibiting the efflux pumps. From a mechanistic point of view, sucrose esters, especially those bearing a longer hydrocarbon chain, were able to fluidize the plasma membrane of epithelial cells at low concentrations, and they could alter the function but not the visible morphology of cellular junctions since no relevant changes were observed in the distribution of junctional proteins as claudin-1, zonula occludens-1 (ZO-1), and β-catenin according to immunostaining [67]. A reversible TEER decrease, induced by the exposure of a compound on mucosa cells or tissues, is indicative of its effect on the reversible modulation of tight junction opening, without any permanent perturbation of membrane properties in terms of structural integrity. The concentration-dependent effect of sucrose C12 on TEER reduction across Caco-2 cells in the range of 0–2 mM was confirmed in another study, even though the release of lactate dehydrogenase (LDH) as a marker for induced cytotoxicity was observed, starting from the concentration of 0.1 mM [68]. The sucrose C6 monoester was tested as a potential permeability enhancer across intestine ex vivo by using a Ussing chamber apparatus. TEER reduction and the apparent permeability coefficients (P_app_) of [^14^C]-mannitol across isolated rat colonic mucosae were determined for this amphiphile in comparison to other common surfactants up to a concentration of 10 mg/mL. At the concentration of 5 mg/mL and 10 mg/mL, sucrose C6 monoester demonstrated to be a good permeability enhancer agent by exerting an effect similar to that of bile salts [69]. Some studies were also carried out in vivo on the intestines of rats using an in situ instillation method. Among different sucrose esters, sucrose C12 (L-1695) was the one causing the most significantly enhanced adsorption of alendronate across both the small and large intestine, as evaluated in vivo using the closed loop method. The observed increased in adsorption was concentration-dependent, without LDH release up to a concentration of 1% *w*/*v*, demonstrating its safe use. Moreover, this study reported the effect of sucrose C12 in promoting membrane fluidity and tight junction opening by providing further evidence concerning the possible effects of this amphiphile in terms of permeability via the transcellular or paracellular routes. Specifically, mechanistic studies using fluorescence anisotropy indicated that sucrose esters can affect the fluidity of a rat small intestine membrane. Moreover, decreased expression levels of claudin-1 and claudin-4 tight junction proteins were found after treatment on a Caco-2 cell, which was used as a reference [70]. Yamamoto et al. also investigated the permeability-enhancing activity in vivo on a rat intestine for different sucrose esters, including sucrose C12 (L-1695), and used 5(6)-carboxyfluorescein (CF) as the model drug. The calculated enhancement ratio for CF across a rat small intestine was around 5 for both sucrose stearate and sucrose C12 when administered at the dose of 1% *w*/*v* [71]. The effect of sucrose C12 in increasing drug permeability across the small intestine using fluorescein isothiocyanate-labeled dextran with a molecular weight of 4000 (FD-4) was also reported. This compound achieved a concentration of 10% *w*/*v*, an effect comparable to that of sodium caprate [72]. A more comprehensive study was performed by McCartney et al., in which the intestinal permeation ability of sucrose C12 (D1216) was assessed in vitro on Caco-2 monolayers, ex vivo on isolated rat intestinal mucosae, and in vivo by rat intestinal instillations. A concentration of 1 mM was able to reduce the TEER below 20% of the initial value and to increase the P_app_ of [^14^C]-mannitol in a significant manner on a Caco-2 cell monolayer. However, at this concentration (1 mM), some cytotoxic effects could also appear as suggested by the non-recovered TEER to initial values after the treatment by the decrease in cell survival to 31% after 24 h of exposure according to the MTS assay, and the alteration in the continuity of tight junction protein between cells. A comparable TEER decrease (below 20% of the initial value) was achieved ex vivo on rat colonic mucosae using a concentration of 5 mM. This concentration also caused a significant increase in the P_app_ of [^14^C]-mannitol and FD-4. Instillations inside jejunum and colon were performed at sucrose laurate concentrations of 50 mM and 100 mM in association with insulin. The obtained blood glucose reduction, when sucrose C12 was used at a concentration of 100 mM, was comparable to that achieved in the presence of sodium caprate as a permeability enhancer for insulin, without remarkable tissue damage [73]. From all these studies, sucrose esters and mainly sucrose laurate (as a mono or a polyester) have emerged as interesting candidates for the development of effective drug permeability enhancers for the intestinal route.

More recently, research has addressed lactose esters as a potential class of drug permeability enhancers across mucosa. The available studies have focused on the in vitro and ex vivo TEER reduction and increased permeability of different saturated and unsaturated lactose esters. Until now, no in vivo permeability studies have been performed on this class of compounds. The aim was to compare the performances of lactose esters with those of sucrose esters and to understand whether the disaccharide moiety is required for the permeability-enhancing effect. A series of lactose-based monoesters bearing saturated C10, C12, C14, or C16 acyl chains was synthesized and tested in terms of cytotoxicity and TEER decrease in intestinal Caco-2 and airway epithelium Calu-3 cells. This study demonstrated the low cytotoxicity potential of these compounds toward the tested cell lines, since the IC_50_ (inhibitory concentration causing 50% of cell death) values, calculated from MTS and LDH assays, were slightly higher than the corresponding CMC values. Therefore, from a cytotoxicity point of view, the amphiphiles here considered can be defined as having a “mild behavior”, which makes them attractive for pharmaceutical use. With regard to the effect on TEER, lactose C12 was the surfactant showing the best performance, since it was able to decrease TEER at a non-cytotoxic concentration (0.476 mM) to the 25% of the baseline value on Calu-3 cells in a reversible manner. On the contrary, lower TEER reductions were observed for the other compounds with a different acyl chain length. Overall, the results obtained in this study are supportive for the possible use of lactose C12 as a permeability enhancer agent [43]. Unsaturated 6′-*O*-lactose-based monoesters derived from palmitoleic acid (C16:1), oleic acid (C18:1), and nervonic acid (C24:1) have also been synthesized and tested for TEER and permeability across Caco-2 cells [42,44]. In both studies, surfactant concentrations lower than the calculated IC_50_ values were applied on Caco-2 cell for TEER assay and a concentration-dependent decrease was observed. The maximum decrease in TEER for lactose C16:1 and lactose C24:1 was achieved at a concentration of 0.1 mg/mL after 2.5–3 h from the treatment. Lactose C24:1 was demonstrated to be more effective since the decrease in TEER was in the range 41–65% with respect to the baseline value in comparison to lactose C16:1, which induced, at the same experimental conditions, a decrease in TEER in the range of 62–68% [42]. For the lactose C18:1, the TEER values recorded between 2 and 3 h from application on a cell monolayer were around 50% at a concentration of 0.125 mg/mL, and around 40% at a concentration of 0.25 mg/mL with respect to the baseline value. In all tests, TEER recovered after 24 h from the removal of surfactants, indicating the reversible effect of these compounds on tight junctions opening. These results configure the tested unsaturated 6′-lactose monoesters as compounds having a moderate and reversible effect on TEER (measured in vitro on cell lines) at non-cytotoxic concentrations [44]. The permeability-enhancing effect of lactose C16:1 and lactose C24:1 was investigated across the Caco-2 cell monolayer using fluorescein isothiocyanate-labeled ovalbumin (FITC-OVA) (45 kDa) as a model protein. The higher P_app_ values were observed at a concentration of 0.2 mg/mL for lactose C16:1 (11.5-fold-increase of permeability with the respect to control) and at a concentration of 0.1 mg/mL for lactose C24:1 (2.5-fold increase in permeability) [42]. On the other side, the permeability-enhancing performances of the lactose C18:1 monoester was tested on a Caco-2 cell monolayer using fluorescein isothiocyanate-labeled dextran 4 kDa (FD4) as the model protein. The maximum effect was observed at the highest tested concentration of 0.25 mg/mL, which determined an increase in FD4 permeability of around 7.7-fold with respect to the control [44]. The permeability-enhancing effect of lactose C12 on isolated rat colon mucosae in comparison to commercial sucrose C12 and the synthesized trehalose C12 monoester used as the monosaccharide-based surfactant control was reported by McCartney et al. [53]. For all three laurate monoesters, the apical concentrations between 0.5 mM and 8 mM were applied to rat colon mucosa using a Ussing chamber apparatus, and TEER changes together with the [^14^C]-mannitol permeability were monitored over time (up to 2 h). Overall, TEER values decreased over time in a concentration-dependent manner. Sucrose C12 and lactose C12 monoesters showed a decrease in TEER in the range of 30–45% with respect to the control value in the range of concentrations from 1 mM to 4 mM. A further decrease in TEER occurred at the concentration of 8 mM, down to 20% of the control value. The effect exerted by trehalose C12 was less pronounced instead, since TEER did not decrease to 30% of the control value, even at the highest tested concentration of 8 mg/mL. The observed [^14^C]-mannitol permeability reflected the results obtained from the TEER assay. Indeed, the calculate P_app_ values were much higher for sucrose C12 and lactose C12 monoesters than for trehalose C12 monoester. At the tested concentration of 8 mM, the P_app_ increase was 5.4-fold for lactose C12, 8.8-fold for sucrose C12, and 3.2-fold for trehalose C12 with respect to the control. The larger effect in permeability for sucrose C12 in comparison to lactose C12 at 8 mM could be influenced by tissue damage, evidenced from the histological analysis, which was observed for colonic mucosae exposed to sucrose C12 but not for mucosae exposed to the same concentration of lactose C12. It is worth noting that at a lower concentration (4 mM), for which no tissue damage was observed for both compounds, the measured increase in permeability was 3.1–3.6-fold for sucrose C12 and 2.8–3.7-fold for lactose C12. In agreement with these results, a comparable performance of lactose C12 and sucrose C12 can be argued, at least on the basis of intestinal permeability studies performed ex vivo. The presence of a disaccharide moiety (as in sucrose or lactose) could be crucial for the permeability-enhancing effect of this class of compounds, but further studies are required since the only tested monosaccharide was trehalose C12 in only one study. Mechanistic studies regarding the mode of action of lactose esters on membrane fluidization and tight junction expression are still lacking in the literature and are encouraged in the future.

Both sucrose and lactose esters (especially the C12 derivative) demonstrated good permeability-enhancing ability on intestinal models, at least under the tested conditions reported in the available literature. However, the development of these compounds as “real” permeability-enhancing agents usable in formulations requires dditional in vivo studies. This would confirm current available evidence and provide more detailed information that can elucidate the molecular mechanism of the observed permeability effects.

## 6. Antimicrobial Activity

The extensive use of antimicrobial agents has had a negative impact on public health, the environment, and the economy. Indeed, although the broad application of antimicrobial substances causes a decrease in microbe contamination, the development of antimicrobial resistance (AMR) is increasingly present and new resistance mechanisms are being developed that are consequently making it difficult to treat common infectious diseases [74,75]. For the past 70 years, infectious diseases have been treated with conventional antimicrobial chemotherapy, but the effectiveness of these therapies is constantly decreasing due to the occurrence of AMR microorganisms, and there are currently no effective alternatives. Therefore, research is increasingly engaged in the discovery of new substances with antimicrobial activity. In some cases, however, significant therapeutic results were not obtained due to poor penetrability and water solubility [76].

In this context, in recent years, sugar fatty acids esters have been synthesized and tested for their antimicrobial and antifungal activity against food-borne pathogens and fungi, as summarized in Table 1.

The small library of the examined glucose-based esters, including glucose C8, C10, C12, C14, and C16, has clearly demonstrated that the antibacterial activity decreased with the increasing length of the fatty acid-saturated chain. Indeed, MICs of 256 µg/mL against the examined bacteria and fungi were observed for glucose C8 and C10 (with the exception of *Klebsiella pneumonia*, *Pseudomonas aeruginosa* and *Staphylococcus aureus* with MIC > 256 µg/mL in the case of glucose C8), while MICs > 256 µg/mL were obtained with glucose C12, C14, and C16 [23]. A quite similar behavior was observed with the series of mannose-based esters for which MIC values of 256 µg/mL against bacteria and fungi were registered, while higher MICs (in many cases >256 µg/mL) were shown for mannose C8, C14, and C16 [16]. With regard the aromatic series, including lactose phenylacetate, lactose *p*-phenyl benzoate, lactose *p*-biphenyl acetate, and lactose *p*-triphenyl benzoate, the MIC values were 256 µg/mL against bacteria and fungi and only in two cases (*P. aeruginosa* and *S. aureus*) MICs > 256 µg/mL were obtained with lactose phenylacetate.

The sucrose-based esters C12 have a Minimum Inhibitory Concentration (MIC) ranging from 256 to 1024 µg/mL for bacteria and 128 to 256 µg/mL for fungi [46]. Our data agree with those of Liang et al. [25], who report weak or moderate inhibitory activity of lactose sugar esters with MICs in the range of 64 to 512 µg/mL. On the contrary, in the research of Lee et al., the MIC values of lactose esters have not been determined, and, for most of the tested microorganisms, the reported values are very high (from <0.05 to 5 mg/mL) [77]. This trend was also observed with lactose-based esters, which were shown to possess antimicrobial activity, with MIC values ranging from 64 to 128 µg/mL in the case of lactose C16:1, lactose C24:1 [42], and lactose C18:1 [44], while higher MICs were observed for lactose C8 and C10, up to a negligible antibacterial activity (MIC > 256 µg/mL) for lactose C12, C14, and C16 [23]. On the other hand, all these compounds showed interesting antifungal activity, with MICs ranging from 128 to 256 µg/mL against *C. albicans* ATCC 10231 [16]. The last three lactose-based esters demonstrated wide antifungal activity (MIC from 128 to 512 µg/mL) rather than antibacterial activity (MIC from 128 µg/mL up to >1024 µg/mL) [46,78].

**Table 1:** Antibacterial and antifungal activity of the synthetized SBEs tested against different strains of gram-positive (*S. aureus*, *E. faecalis*, *L. monocytogenes*), gram-negative (*E. coli*, *K. pneumoniae*, *P. aeruginosa*, *S. enteritidis*, *Y. enterocolitica*), as well as against mycetes and fungi (*C. albicans*, *T. mentagrophytes*, *T. rubrum*, *T. violaceum*, *Epidermophyton floccosum*, *A. fumigatus*, *A. niger*, *Fusarium* spp.) in the recent years. Data are expressed as MIC (µg/mL).

SBEs	MIC (µg/mL)	Microorganisms		Reference
		Bacteria	Fungi	
**Monosaccharides**				
Glucose C8	256 or >256	*E. coli* O157:H7 ATCC 35150, *K. pneumoniae* ATCC 13833, *P. aeruginosa* ATCC 9027, *S. enteritidis* ATCC 13076, *E. faecalis* ATCC 29212, *L. monocytogenes* ATCC 7644, *S. aureus* ATCC 43387	*C. albicans* ATCC 10231	[23]
Glucose C10	256			
Glucose C12	>256			
Glucose C14	>256			
Glucose C16	>256			
Mannose C8	>256			
Mannose C10	256			
Mannose C12	256			
Mannose C14	256 or >256			
Mannose C16	256 or >256			
**Sucrose**				
Sucrose C12	128–1024	*S. aureus*, MRSA and *P. aeruginosa* strains	*T. mentagrophytes*, *T. rubrum*, *T. violaceum*, *E. floccosum*, *C. albicans* ATCC 10231	[46]
Sucrose C8	1024 or >1024	*E. faecalis* ATCC 29212, *E. coli* O157:H7 ATCC 35150, *S. aureus* ATCC 29213 and ATCC 43300, *K. pneumoniae* ATCC 13883, *L. monocytogenes* ATCC 7644, *S. enteritidis* ATCC 13076, *P. aeruginosa* ATCC 9027	*A. fumigatus* IDRAH01, *A. niger* ATCC 9642, *Fusarium* spp., *C. albicans* ATCC 10231	[33]
Sucrose C11:1	512 to >1024			
Sucrose undecylinate	1024 or >1024			
Sucrose C16:1	16 to >1024			
Sucrose C18:1	16 to >1024			
Sucrose C18:2	32 to >1024			
Sucrose C18:3	128 to >1024			
Sucrose C24:1	1024 or >1024			
Sucrose benzoate	256 to >1024			
Sucrose phenylacetate	256 to >1024			
Sucrose *p*-phenylbenzoate	1024 or >1024			
Sucrose *p*-biphenylacetate	1024 or >1024			
Sucrose *p*-terphenylacetate	>1024			
**Lactose**				
Lactose C8	256	*E. coli* O157:H7 ATCC 35150, *K. pneumoniae* ATCC 13833, *P. aeruginosa* ATCC 9027, *S. enteritidis* ATCC 13076, *E. faecalis* ATCC 29212, *L. monocytogenes* ATCC 7644, *S. aureus* ATCC 43387	*C. albicans* ATCC 10231	[23]
Lactose C10	128–256			
Lactose C12	256 or >256			
Lactose C14	256 or >256			
Lactose C16	256 or >256			
Lactose phenylacetate	256 or >256			
Lactose *p*-phenylbenzoate	256			
Lactose *p*-biphenylacetate	256			
Lactose *p*-terphenylacetate	256			
Lactose C16:1 Lactose C24:1	64–128	*E. coli* O157:H7 ATCC 35150, *L. monocytogenes* ATCC 7644, *S. enteritidis* ATCC 13076, *E. faecalis* ATCC 29212, *S. aureus* ATCC 43387, *P. aeruginosa* ATCC 9027, *Y. enterocolitica* ATCC 27729	*C. albicans* ATCC 10231	[42]
Lactose C18:1	128			[44]
Lactose C11:1	512 to >1024	*S. aureus*, MRSA and *P. aeruginosa* strains	*T. mentagrophytes*, *T. rubrum*, *T. violaceum*, *E. floccosum*, *C. albicans* ATCC 10231	[78]
Lactose C18:2 Lactose C18:3	128 to >1024			[46]

The antimicrobial activity of other sugar fatty acid esters was reported in the literature. Ferrer et al. [79] reported that 6-*O*-lauroylsucrose and 6′-*O*-palmitoylmaltose showed activity against Gram-positive and Gram-negative bacteria, and yeasts. Similarly, Zhao et al. [80] investigated the activity of sucrose fatty acid esters against five food-related bacteria (*Bacillus cereus*, *Bacillus subtilis*, *S. aureus*, *E. coli*, *Salmonella typhimurium*), but no action was observed for sucrose monostearate, and a weak action for sucrose C14. On the contrary, sucrose C10 demonstrated the strongest antimicrobial activity, particularly against Gram-positive bacteria. A similar trend was observed by Tabisz et al. [81], who reported better antimicrobial activity for sucralose C8 in comparison to esters with longer aliphatic side chains, showing weak (sucralose C14) or no (sucralose C18) antimicrobial effect. Karlová et al. [82] also indicated that the antibacterial activity of fructose fatty acid esters decreased as the length of the fatty acid increased.

A very recent promising tool in biological applications is the possibility that some of these surfactants counteract the formation of bacterial biofilms. As already known, this kind of microbial cell organization is more resistant to antibiotics and some physical treatments [83,84], thus representing a serious problem for infectious disease outbreaks. The biofilm formation is a dynamic process, involving at least four stages: planktonic, reversible, and irreversible attachment, maturation, and dispersion [85], each regulated by a different gene expression. One of the possible approaches to controlling biofilm is to limit or inhibit its formation at an early stage by molecules with antibiofilm activity. As reported by Campana et al. [23], the antibiofilm activity of glucose C10, mannose C10, lactose C10, and lactose *p*-biphenyl acetate was assessed at different stages of the biofilm development (24 h, 48 h and 5 days) of four representative food-borne pathogens (*E. coli* O157:H7 ATCC 35150, *L. monocytogenes* ATCC 7644, *S. aureus* ATCC 43387, and *S. enteritidis* ATCC 13076). The antibiofilm effect resulted in increased percentages from 24 h to 96 h, as observed for *E. coli* O157:H7 (66.7% and 97.2%, respectively) and *L. monocytogenes* (62.3% and 91.8%); for *S. enteritidis*, the highest percentages of biofilm reduction were observed in the shorter formation time (98.5%, 24 h), while for *S. aureus*, it was observed in the intermediate period (99.9%, 48 h). On the contrary, glucose C10 and lactose C10 were unable to limit biofilm formation after 24 h and 48 h (with the only exception being *S. enteritidis* ATCC 13076), reaching percentages >30% (from 34.6% to 55.3%) in the long period of biofilm development [23].

## 7. Conclusions

SBEs are a broad group of biocompatible and biodegradable compounds with established and potential future applications in the pharmaceutical, biomedical, cosmetic, and food industries. Regioselective acylation at the C6-position of disaccharides (e.g., sucrose, lactose) and monosaccharides (e.g., glucose, mannose) can be effectively achieved via an enzymatic reaction using immobilized lipases, which are able to operate at different experimental conditions (pH, temperature, and solvent). Alternative synthetic approaches are represented by the Mitsunobu reaction and the use of aluminosilicate minerals as Lewis’s acid catalysts. With regard to the physicochemical properties, all SBEs possess good surface activity, being influenced by both the sugar moiety and the length of the hydrocarbon chain. Specifically, lower CMC values were generally found for monosaccharide monoesters with respect to the corresponding disaccharide derivatives. Foaming and emulsifying activity were found to be mainly affected by the hydrophobic portion, being overall higher for medium-chain (C12 and C14) and long-chain derivatives (≥C14), respectively. The use of these amphiphilic compounds as permeability enhancers or as antimicrobial agents represents an attractive field of research and application. The available studies on in vitro and ex vivo models support the potential development of these compounds as effective permeability enhancers for drugs and biological compounds across the mucosa, although other in vivo investigations are necessary to assess their efficacy under real-world conditions and to further elucidate their mechanisms of action. The available results regarding antimicrobial and antibiofilm activity are also promising for their use as preservatives and possibly disinfectants. Overall, the scientific information on SBEs reported in the review supports the further development of this class of biocompatible and biodegradable amphiphiles in different technological fields.

## Data Availability

Data is contained within article.
